# The Foreign Journals: Anatomy and Physiology

**Published:** 1840-04

**Authors:** 


### PART THIRD.

### Selections from the British and Foreign Journals.

### I. THE FOREIGN JOURNALS.

### ANATOMY AND PHYSIOLOGY.

*On the Injurious Qualities of Menstrual Blood, and on its Probable Causes.*
By Dr. REMAK, of Berlin.

THIS paper is chiefly useful for the information it affords respecting the composition of a fluid which few have either the inclination or the liberty to examine. The author adduces but little fresh evidence in support of the universal popular belief that menstrual blood can produce urethral discharge or other affections of the male genital organs; but he thinks that the occasional occurrence of these symptoms, of which he has no doubt, depends on the condition, not of the blood, but of the mucus with which it is mixed.

By microscopical examination of the menstrual fluid under various circumstances, he found that whenever it had a red colour, it contained blood-globules, and that the intensity of its colour depended on their number. At the beginning and towards the end of its flowing, when it is pale and whitish, it contains a preponderating quantity of laminæ of epithelium and of mucus-corpuscles, but their quantity becomes proportionally less and less as the fluid presents more blood-globules and a deeper colour. The author believes that this mucus which is constantly present in the menstrual fluid, may, like the secretions of all mucous membranes when in a morbid state, possess the power of producing a similar morbid state in other membranes of the same kind. He relates a case which confirms this idea: a very obstinate gonorrhea, in which the menstrual fluid of the only female with whom the patient had connexion, and in whom there was not the least probability of gonorrhea existing, contained an abundance of thin pearl-coloured mucus. Connexion did not produce the gonorrheal symptoms except when the female was menstruating.

*Medicinische Zeitung. Dec.* 25, 1839.

*Evolution of Heat in Plants.* By MM. VROLIK and VRIESE.

SOME additional experiments on *colocasia odora* (see B. and F. M. R., Vol. IV., p. 25,) have been recently made by MM. Vrolik and Vriese. Besides affording a complete confirmation of the results obtained by others, they have established some new and important facts. A flowering spadix placed in oxygen was compared with a corresponding one in atmospheric air. Within half an hour after its immersion, its temperature rose 2·7° C. (4·9° F.), whilst that of the one in air was falling 1·1° C. (2° F.) During and after the emission of the pollen in both, the maximum temperatures were the following:

| | | | | | |
|---|---|---|---|---|---|
| Spadix in Oxygen . | 30·6° C. | 87·1° F. | Spadix in Atm. Air . | 27·8° C. | 82° F. |
| Surrounding Temp. . | 23·3° C. | 74·0° F. | Surrounding Temp. . | 22·8° C. | 73° F. |
| Difference . . . | 7·3° C. | 13·1° F. | Difference . . . | 5·0° C. | 9° F. |

The temperature of the spadix in oxygen rose much more rapidly than that of the other; so that early in the day the difference was 4·6° C; that of the surrounding media being ·3° in favour of the lowest, so as to make the difference really 4·9° C., or 8·8° F., whilst that of the maxima of both was 2·3° C., or 4·1° F. In these experiments, a large quantity of oxygen was observed to disappear; on the other hand carbonic acid was liberated, which, being absorbed into the water below, caused it to rise into the vessel.

A spadix beginning to flower was placed in nitrogen. It had already acquired an elevation of temperature to the amount of 2·6° C. This gradually diminished, although the expansion of the flower continued. At the time of the emission of the pollen there was no elevation; whilst a corresponding spadix in the open air showed an elevation of 8·6° C. No change in the bulk of the confined gas was noticed in this experiment.

These experiments afford a striking confirmation of the doctrine now rapidly gaining ground amongst physiologists, that the evolution of caloric from living beings is dependent upon certain organic processes, in which the combination of oxygen (either immediately or remotely derived from without) and the carbon of the system are principally concerned. These go on most actively during the period of inflorescence; but they are not restricted to it. The same process of *respiration* is constantly going on in almost every part of the living plant; but, owing to its feeble amount, and the extended surfaces by which the caloric is dissipated as fast as it is generated, it is difficult to acquire any evidence of its liberation.

By the use of the thermo-multiplier, however, which was so successfully employed by MM. Becquerel and Breschet in their experiments upon animal temperature, M. Dutrochet has succeeded in proving the existence of a *proper heat* in plants, more especially in the green parts, which are the seat of the more active organic changes. In order to obtain unequivocal evidence to this effect, it was necessary to devise means of excluding the other causes which produce an elevation of temperature in some parts of the vegetable structure. Thus, in a growing tree, the sap which rises in spring, being principally drawn from a depth of two or three feet in the soil, will be warmer, perhaps even by several degrees, than the atmosphere. On the other hand, the evaporation constantly going on from the soft and moist surfaces of rapidly-growing parts, will depress their temperature. M. Dutrochet employed in his experiments plants either growing in pots, or cut off and placed in water of the same temperature as the surrounding air; and he did not rely upon the temperature of the stem, but upon that of the leaves and young shoots, in reaching which the sap would have acquired the temperature of the plant. He thus avoided the first source of error. The second was prevented by making his comparison, not between these parts in a growing plant and the surrounding atmosphere, but between corresponding parts in a living plant, and in one recently killed by immersion in hot water, and therefore susceptible of the diminution of temperature which evaporation causes, to the same degree as the living plant. The experiment was in some instances still further prevented from error by the immersion of both plants in an atmosphere saturated with aqueous vapour. With these precautions the result was *constantly* the same. An elevation of temperature to the amount of one fourth or even one third of a degree (Cent.) was observed in the herbaceous parts of actively-growing plants, differing with the species, the activity of vegetation, and the period of the day. There is a sort of diurnal paroxysm, which generally manifests itself about noon; in some species an hour before, in others an hour after. The *proper heat* increases up to this period, attains its maximum, and then diminishes. The access of this paroxysm depends in part upon the presence of light; but it will occur, though in a diminished degree, for two or three days in a plant withdrawn from the influence of that agent.

*Annales des Sciences Naturelles,* N. S. Botan. Tom. xi.-xii.

### *Experiments on the Motor and Sensitive Roots of the Nerves.* By Dr. Kronenberg, of Moscow.

During my stay in Paris in July (1839), Prof. Magendie had the kindness to show me his recent experiments on the nerves, which he had communicated to the Academy. These, as made in my presence, I will here detail, in the first place, and then notice my own experiments made some time afterwards.

1. M. Magendie laid bare the facial nerve of a dog near its origin and pricked it; at each prick a demonstration of pain was given. The nerve was then cut across, not far from its junction with the fifth pair. Pricking and pinching the cerebral end of the divided nerve produced no indication of sensibility; but pinching the other end obviously caused pain. It appears from this experiment that the sensibility of the facial nerve, as well after as before its union with the fifth pair, depends on this last, and not from any other anastomosis or the influence of the brain sent direct through its origin. M. Magendie then laid bare the roots of the lumbar nerves of a dog. The posterior roots were found as usual very sensible; the anterior were less so, still the dog whined and gave evident signs of pain each time they were pinched. M. M. then divided the motor root; pinching the spinal extremity of this produced no pain, but pinching the other (before its union with the sensitive root) caused the dog to cry out.

2. Some weeks after this I performed the following experiments on rabbits: The facial nerve before its union with the fifth was found sensible, at one time more so, at another less; on dividing it before its union, pinching its under (peripheral) end produced pain, yet not always. I then laid bare the lumbar portion of the spinal marrow, and found the motor roots sensible, but much less so than the sensitive. The following experiments, however, prove that the sensibility of the motor roots is not derived through fibres coming directly from the spinal marrow, but is dependent on the other (the sensitive) roots. When I stimulated the motor root, the sensitive root being undivided, pain was evinced, but when the latter was divided the same stimulus produced no sensation. Magendie's experiment equally proved this, and its repetition by myself confirmed the same. After division of the anterior root, the posterior being untouched, the under or peripheral end was always sensible, the upper not. The case is similar with the anterior column of the spinal marrow—pain being only produced when the posterior roots were uninjured.

In order to settle this point still more firmly, and to ascertain the course of the fibres, I made the following experiment: I made in the angle of union of the two roots a small incision, about half a line in extent, leaving both the roots untouched: it was then found that the same phenomena no longer took place; thus, the anterior root and the anterior column of the spinal marrow were now insensible, and on the division of the root both its ends were equally insensible.

This simple and easy experiment proves, first, that a portion of the fibres of the sensitive root extends to the point of union and is reflected back to the anterior column of the spinal marrow; and, secondly, that the return or reflection of the fibres takes place near the point of junction of the two roots.

[These experiments afford a satisfactory explanation of phenomena which have been very perplexing to physiologists. It has been almost constantly observed that some degree of sensibility appeared to exist in what Sir C. Bell regarded as exclusively the *motor* roots of the nerves; this sensibility being manifested by expressions of pain on the part of the animal when they were irritated. The sensibility of the portio dura has long been known, and was correctly attributed by Sir C. Bell to its reception of filaments from the fifth pair. A similar mixture of the filaments of the posterior roots of the spinal nerves with those of the anterior—these filaments passing *towards* the centre as well as *from* it—may be reasonably anticipated; and this supposition fully explains all the phenomena which have led Magendie and others to the opinion that the anterior roots are sensible. The supposed motor properties of the posterior roots are fully accounted for by Dr. Hall's discoveries; since irritation of these will produce reflex actions through the motor nerves distributed to the same parts.]

*Müller's Archiv.* Heft. v., 1839.

### *On the Nerves of the Cornea.* By Dr. Pappenheim.

The cornea has been represented by M. Hippolyte Cloquet to be devoid of nerves as well as of blood-vessels. In 1830, however, Schlemm succeeded in tracing nerves as far as the margin of the cornea, though its density prevented his following them between its laminæ. The investigation thus commenced has been pursued by M. Pappenheim, who by a simple process, that of immersing the cornea in acetic acid, or a solution of caustic potass, has been able to trace nerves from the sclerotica into the substance of the cornea. That the nerves thus traced really belong to the cornea, the following facts appear to prove: 1. If the corneal conjunctiva is removed, the nervous filaments are seen on the inner, not on the outer surface of the corneal epithelium. 2. The removal of the iris and membrane of the aqueous humour makes no difference as to the ease with which the nerves may be seen. 3. The nerves are distinctly visible entering the margin of the cornea, but less so towards its centre, where they are ultimately lost between the laminæ.

The nerves may be distinguished from the folds of the choroid which mark the cornea by being colourless, and of uniform thickness; from the fibres of the cornea by being smaller, more superficial, scattered, and arranged in plexuses. Internally they are covered by the membrane of the aqueous humour, externally by the fibres of the cornea. The readiest way of discovering them is to immerse the dissected cornea in water, placing it between two plates of glass, with its inner surface turned upwards—gentle pressure, and the light of a lamp are required, and at first a slightly magnifying lens will be desirable. The nerves will then be seen taking the course of the blood-vessels, and composed of fasciculi for the most part separate.

*Monatschrift für Medicin, &c. June,* 1839.

